# Sc-CO_2_ extraction of fish and fish by-products in the production of fish oil and enzyme

**DOI:** 10.1186/s40643-022-00509-3

**Published:** 2022-03-12

**Authors:** Nur Anati Jamalluddin, Normah Ismail, Siti Roha Ab. Mutalib, Adi Md Sikin

**Affiliations:** Department of Food Science and Technology, Faculty of Applied Sciences, Universiti Teknologi MARA (UiTM), 40450 Shah Alam, Selangor D.E Malaysia

**Keywords:** Sc-CO_2_, Digestive enzymes, Fish by-products, Defatted, Protein, Fish oil

## Abstract

Supercritical carbon dioxide (Sc-CO_2_) is an alternative tool to extract lipid for the production of fish oil and enzyme from fish by-products (FBPs). In the application of Sc-CO_2_, this review covers sample preparation, lipid extraction operation, and characterization of fish oil and enzyme as final products. Generally, the fish samples with moisture content less than 20% and particle size less than 5 mm are considered before lipid extraction with Sc-CO_2_. Sc-CO_2_ parameters, such as pressure (P), temperature (T), extraction time (t_ext_), and flow rate (F), for simultaneous recovery of fish oil, protein, and enzyme were found to be less severe (*P*: 10.3–25 MPa; *T*: 25–45 °C, *t*_ext_: 20–150 min; *F*: 3–50 g/min) than the extraction of fish oil alone (*P*: 10–40 Mpa; *T*: 35–80 °C; *t*_ext_: 30–360 min; *F*: 1–3000 g/min). The enzyme from the Sc-CO_2_ defatted sample showed higher activity up to 45 U/mg due to lower denaturation of protein as compared to the organic solvent treated sample albeit both samples having similar pH (6–10) and temperature stability (20–60 °C). Overall, mild extraction of lipid from FBPs using Sc-CO_2_ is effective for the production of enzymes suitable in various industrial applications. Also, fish oil as a result of extraction can be produced as a health product with high polyunsaturated fatty acids (PUFAs) and low contamination of heavy metals.

## Introduction

The growing population has urged the fish industry to produce more fish products to fulfil consumers’ demands and diets. From 1961 to 2018, the food fish consumption per capita has grown by about 1.5% per year (FAO 2020), generating tonnes of fish by-products (FBPs). To illustrate, 75.24 million metric tonnes (MMT) of waste is generated from the total fish production of 167.2 MMT globally, which is equivalent to 45% of the live weight (Murthy et al. 2018). FBP is usually composed of heads (9–12% of total fish weight), viscera (12–18%), skin (1–3%), bones (9–15%), and scales (about 5%) (FAO 2020). It is often regarded as waste and discarded without any attempt of recovery. FBPs are normally used as fertilizers and silage as well as feed for aquaculture livestock and animals or pets. However, inappropriate disposal of FBPs creates an unhygienic environmental atmosphere (Mohanty et al. 2018). Nevertheless, FBP is a great source for the recovery of polyunsaturated fatty acids (PUFAs), proteins, enzymes, minerals, and other bioactive compounds with functional properties. For example, fish viscera are rich in digestive enzymes, especially protease (e.g. pepsin, cathepsin, trypsin, chymotrypsin, calpain, and collagenase)(Haard and Simpson 1994; Shahidi and Kamil 2001). Protease breaks down proteins into smaller polypeptides or single amino acids (Oyeleke and Auta 2010) and is mainly derived from animal, plant, and microbial sources (Chaijaroen 2015). Protease dominates half of the world industrial enzyme market with various applications, such as food, detergent, leather, pharmaceuticals, cosmetics, silk degumming, silver recovery, chemical industry, and waste management (Kumari et al. 2015; Naveed et al. 2021). Generally, the global enzyme market is expected to grow from $5.01 billion in 2016 to $6.32 billion in 2021 as according to Business Communication Company (BCC) Research, with market trends predicting a shift towards increased technical enzyme production (Dewan 2017).

Many issues have been addressed in the production of protease derived mainly from animal and plant as mentioned by Guerrand (2018) and Gurumallesh et al. (2019). For instance, the development of animal and plant-derived proteases is still limited due to the scarcity of sources. These enzymes are also challenging to maintain the batch-to-batch consistency in the production of this enzyme. Protease derived from animal faces a scrutiny concerning ethical and religious issues. Next to that, the enzyme source related to Bovine Spongiform Encephalopathy (BSE) diseases is a major safety concern by the consumers. The enzyme produced from microbial sources is not exceptional. The issues emerge from the fermentation process, which are mostly associated with the halal status of growth media, culture for isolation, and processing aids, such as emulsifiers, antifoaming agents, and preservatives (Ermis 2017). Due to these, the demand for fish proteolytic enzymes has been escalating. Other than resolving dumping issues and environmental pollution, trypsin from FBPs is reported to have resembled mammalian trypsin in terms of molecular weight, amino acid composition (22–30 kDa), and sensitivity towards inhibitors (Sriket 2014). Sanromán and Deive (2017) and Shahidi and Kamil (2001) reported that fish proteases from viscera can be a substitute to rennet in the manufacture of cheese, which not only avoids non-conformity to halal, kosher, and vegetarian food requirements but also produces new flavour and texture to the product (Sanromán and Deive 2017).

Previously, several attempts to extract and purify protease from fish and FBPs without Sc-CO_2_, for example, by using tris buffer, ethanol or acetone, ammonium sulphate, dialysis, and several chromatography methods were reported in this study, such as viscera of mussel (*Mytella charruana*) (Dornelles et al. 2018), silver catfish (*Pangasius sutchi*) (Ismail and Jaafar 2018), Indian major carp (*Labeo rohita*) (Geethanjali and Subash 2018), neon flying squid (*Ommastrephes bartramii*) (Zhang et al. 2019), silver carp (*Hypophthalmichthys molitrix*) (Abe et al. 2020), Nile tilapia (*Oreochromis niloticus*) (Bezerra et al. 2005; Kudre and Thongraung 2014; Chaijaroen and Thongruang 2016), Monterey Sardine (*Sardinops sagax caerulea*) (Salazar‐Leyva et al. 2017), hybrid catfish (*Clarias macrocephalus x Clarias gariepinus*) (Klomklao et al. 2010, 2011), and farmed giant catfish (*Pangasianodon gigas*) (Rawdkuen et al. 2012; Ketnawa et al. 2013, 2014; Vannabun et al. 2014). Based on these studies, the protease exhibits high catalytic activity and heat stability over a wide range of temperature (40 to 60 °C) and pH (6 to 11). Unfortunately, the presence of lipids in fish viscera reduces the efficiency of extracting, isolating, and purifying the enzymes (Kishimura and Hayashi 1999; Uddin et al. 2009; Kim and Dewapriya 2014; Asaduzzaman and Chun 2015). Furthermore, the presence of lipids accelerates protein oxidation, resulting in detrimental protein quality (Matsushita et al. 1970; Kanner and Rosenthal 1992).

**Fig.1:**
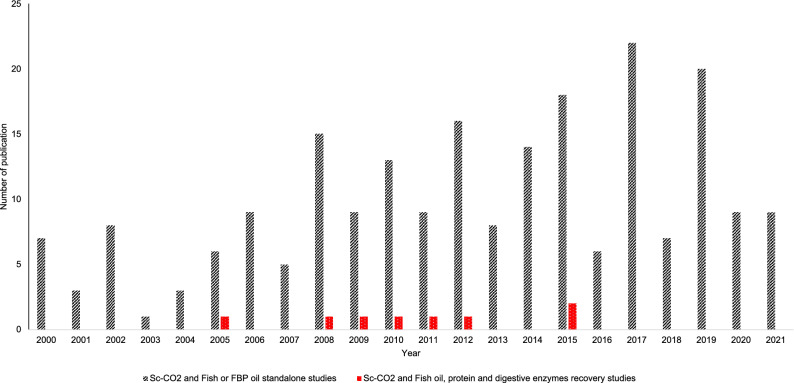
Published journal articles related to Sc-CO_2_, fish oil, recovery of protein, and enzymes from FBP

The profiling of fatty acid for the benefits of PUFAs in fish oil extracted using Sc-CO_2_ is well documented (Sánchez-Camargo et al. 2011, 2012; Sarker et al. 2012; Lisichkov et al. 2014; Hajeb et al. 2015; Ferdosh et al. 2015; Haq et al. 2017; Kuvendziev et al. 2018), but attentions are gradually shifted on lipid extraction for the isolation of enzymes (Uddin et al. 2009; Ali-Nehari et al. 2012; Asaduzzaman and Chun 2015) or collagen (Ahmed et al. 2017). As shown in Fig. 1, the study on lipid removal using Sc-CO_2_ for enzyme recovery is still very scarce although it started as early as 2006. This could be due to insufficient information on handling the delicate fish protein prior to or during Sc-CO_2_ extraction. For example, Park et al. (2006) and Park et al. (2008a) compared the effect of Sc-CO_2_ and organic solvent for lipid extraction on crude enzymes activity and protein properties. Also, the fatty acid profiling was carried out in the oil collected after extraction (Park et al. 2006, 2008; Uddin et al. 2009; Asaduzzaman and Chun 2015). The most recent work on lipid extraction using Sc-CO_2_ for enzyme recovery was reported by Asaduzzaman and Chun (2015) and Lamas (2015). However, the scope of research was expanded from extracting to purifying enzymes from the defatted sample only in the later years (Uddin et al. 2009; Chun et al. 2011; Ali-Nehari et al. 2012; Asaduzzaman and Chun 2015).

This paper reviews the application of Sc-CO_2_ for the purpose of fish oil collection and enzyme purification from fish and FBPs. It covers the effect of fish sample preparation and Sc-CO_2_ extraction on oil yield and enzyme activity. Overall, Sc-CO_2_ as a potential alternative to conventional organic solvent for the extraction of lipid in the production of fish oil and enzyme was examined.

## Significance of lipid removal for enzyme purification

There are several studies that successfully recovered digestive enzymes after removing the lipid layer in the sample via Sc-CO_2_ technology (Park et al. 2008; Uddin et al. 2009; Chun et al. 2011; Ali-Nehari et al. 2012). Intracellular lipid is strongly bound to the protein matrix (Rubio-Rodríguez et al. 2012), which requires its removal from FBPs in order to achieve higher efficiency of enzyme isolation (Asaduzzaman and Chun 2015). For example, Geethanjali and Subash (2013b) claimed that 45% of fat in the viscera of Indian major carp (*Labeo rohita*) (Pathak et al. 1954) makes the homogenization of the sample difficult. Furthermore, the resulting extract contains a high quantity of finely dispersed lipid which complicates the subsequent purification steps of enzymes. Likewise, Bhaskar et al. (2007) found a considerable amount of protease activity and high fat content (> 27%) in the viscera of Indian major carp (*Labeo rohita*). They suggested that it would be more worthwhile to remove lipid from FBPs before attempting the recovery of proteases.

Fish is rich in omega-3 and omega-6 PUFAs which is essential for health maintenance (Arbex et al. 2015), but highly susceptible to lipid peroxidation (Hematyar et al. 2019). This results in the reaction of free radicals, hydroperoxides, and aldehydes with proteins, causing the production of protein-centred free radicals and subsequent protein structural alterations (Kanner and Rosenthal 1992; Hematyar et al. 2019). The protein functional groups located in the amino acid residues side chain and the peptide backbone are an easy target by the reactive oxygen species (ROS) during the oxidation process (Hematyar et al. 2019). The active site of protein molecules can be easily damaged by lipid peroxidation and thus leads to a loss in solubility of protein and enzymatic activity (Matsushita et al. 1970). For example, Matsushita et al. (1970) found out that ribonuclease was largely inhibited by linoleic acid hydroperoxides (LAHPO), but the other enzymes, such as trypsin, chymotrypsin, and pepsin, were inhibited by both linoleic acids and LAHPO. The defatted sample of Sc-CO_2_ tends to preserve longer due to removal of the lipid layer and lowers the risk of oxidation. Enzymes become more stable when they are exposed to Sc-CO_2_ because of their conformationally rigid in the dehydrated state (Budisa and Schulze-Makuch 2014). Therefore, the defatted sample can be stored soundly with minimize risk of deterioration and can be used at any time subsequently for the purpose of extraction, isolation, and/or purification of enzymes.

## Supercritical carbon dioxide as a solvent for lipid extraction

**Fig. 2:**
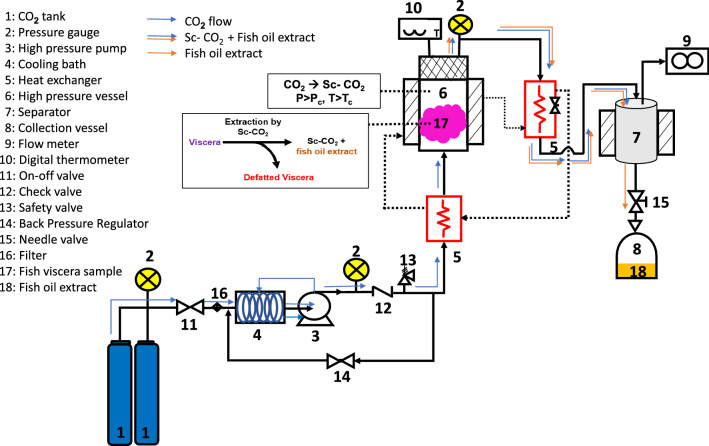
Schematic diagram of the Sc-CO_2_ for the simultaneous production of fish oil and enzyme recovery

Supercritical carbon dioxide (Sc-CO_2_) is a pressurized carbon dioxide (CO_2_) with a moderate critical temperature (31.0 °C) and pressure (7.4 Mpa) (Silva et al. 2020). Figure 2 shows a schematic diagram of the Sc-CO_2_ extraction system for the simultaneous production of fish oil and enzyme. Prior to the Sc-CO_2_ process, the chiller (No.4) is cooled at 4 °C while heating the stainless-steel extraction vessel (No.6) to the desired extraction temperature (*T* > *T*_c_). A high-pressure pump (No. 3) is applied to pump liquid CO_2_ into the extraction vessel for reaching the desired pressure (*P* > *P*_c_). The Sc-CO_2_ as the extraction solvent is then produced at *P* > *P*_c_ and *T* > *T*_c_. It is used to extract less or non-polar compounds, such as lipid layer from the viscera in the form of fish oil extract in the extraction vessel. The Sc-CO_2_ and fish oil extract complex (Sc-CO_2_ + fish oil extract) is then rapidly depressurized by the metering valve (No. 15) to reduce the pressure of CO_2_ before being emitted to the surroundings. The fish oil extract can be collected from the separating vessel (No. 7) and the defatted fish viscera in the extraction vessel is stored appropriately for further use and analysis.

Previous papers gave an overview on new and conventional methods in extracting fish oil (Adeoti and Hawboldt 2014; Khaw et al. 2017; Ivanovs and Blumberga 2017; Ideia et al. 2020). For example, wet pressing is a common extraction method for fish oil at an industrial scale, but it is an energy-intensive process which requires high heat treatment up to 95 °C, centrifugation, and several drying steps followed by pre-staining, pressing of the cooked material, centrifugation to recover the oil from the extract, separation of the press liquor, oil polishing, evaporation of the stickwater, and drying. Likewise, solvent extraction, such as Soxhlet method uses organic solvents, such as methanol, petroleum ether, acetone, chloroform, hexane, or dichloromethane at high temperature (> 78 °C), which is potentially hazardous and requires additional step to remove the solvent by using rotary vapour (Adeoti and Hawboldt 2014). In addition, the extraction could take several hours or even days (Al-Jabari 2002). More importantly, there is residual risk in the finished product due to the incomplete solvent removal after extraction process, makes it unfavourable for consumption (Sarker et al. 2012). These methods affect the nutritional quality of the fish oils, denature proteins, degrades the heat-sensitive, labile, and natural compounds in the samples. On the other hand, Sc-CO_2_ demonstrates great potential in oil extraction because it is non-toxic, non-flammable, inert, and readily available in high purity at low cost (Melgosa et al. 2017). It has tunable physical properties of solvents having liquid and gas-like properties overcoming constraints faced in conventional extraction (Patil 2014; Idris and Markom 2019). Sc-CO_2_ is generally recognized as safe (GRAS) by Food and Drug Administration and European Food Safety Authority (Da Silva et al. 2016; Ferrentino et al. 2019) and thus useful as a substitute for the organic solvent in the defatting process (Chun et al. 2011). Sc-CO_2_ is easily removed from the extract, but most importantly approved for food processing without declaration (Brunner 2005).

Likewise, the capabilities of Sc-CO_2_ can be found in the supercritical fluid chromatography (SFC) separation technique in which Sc-CO_2_ is used as one of the constituents which makes up the mobile phase (Wrona et al. 2019). SFC is used for fish oil refining and omega-3 PUFA concentration with high purity and recovery (Catchpole et al. 2018; Melgosa et al. 2021). However, the industrial scale of SFC requires high initial investment and maintenance costs due to the requirement of specialized stationary phases (Melgosa et al. 2021; Alfio et al. 2021). The columns require special packing material with a particle size ranging from 5 to 10 μm for efficient separation process. Also, the density of the mobile phase in SFC may fluctuate along the chromatographic column due to pressure drop (Catchpole et al. 2018; Johannsen and Brunner 2018). Nevertheless, SFC has found its place in a pharmaceutical company in Spain for the production of highly concentrated omega-3 extracts. They use long chromatographic columns packed with chromatographic silica xerogel, which eliminates the remaining smelly contaminants in the fish oil extracted with Sc-CO_2_ (Ciriminna et al. 2017). Another pharmaceutical company in Germany (KD Pharma) patented SFC technology for the production of omega-3 fish oil with a concentration as high as 99% (Lembke 2011). Most recently, SFC with simulated moving beds (SFC-SMB) enables to fulfil the growing demand for ultrapure eicosapentaenoic acid (EPA) and/or docosahexaenoic acid (DHA) products in pharmaceutical and food industries (Johannsen and Brunner 2018; Melgosa et al. 2021). Although batch SFC process used high quantity of mobile phase, it is less cost intensive than the SFC-SMB process (Johannsen and Brunner 2018). After all, it depends on the intended final products. Sc-CO_2_ without coupling with SFC technique is still considered efficient and feasible to extract both high quality of fish oil and enzyme from fish and/or FBPs.

The industrial implementation of Sc-CO_2_ is indeed hindered by the high initial investment and installation cost. Therefore, fish oil industry maintains the existing solvent extraction using hexane or petroleum ether as the technique is more well known and offers similar extraction yields (Staby and Mollerup 1993; Reverchon and De Marco 2006; Melgosa et al. 2021). Nevertheless, Sc-CO_2_ is expected to have high return for its focus on high-value product (Pereira and Meireles 2007). This is because the end products are perceived as greener, cleaner extract, and more natural by the consumer allowing for higher price tags. Although enzyme extracted from defatted FBPs using Sc-CO_2_ may seem far from the commercialization stage, the aforementioned view should be considered by the enzyme industry to step up their game.

In addition to enzyme, defatted fish meal, which is rich in protein can be obtained as a solvent-free feedstock as a results of lipid extraction using Sc-CO_2_. The feed industry, particularly, will be able to produce healthier feed for pets, animals, and livestock (Melgosa et al. 2021). The protein fraction can be extracted and hydrolysed to obtain fish protein and fish protein hydrolysates with potential bioactive applications (Melgosa et al. 2021). Overall, the ‘zero-waste’ utilization of fish and/or FBPs will be benefited by various industries, such as food, pharmaceuticals, agriculture, and biofuels, as a results of Sc-CO_2_ extraction.

Sc-CO_2_ is selective towards extracting low molecular weight (MW) compounds (< 250 Da) having less polar groups or non-polar compounds (i.e. lipophilic), such as lipids, cholesterol, aldehydes, ethers, esters, and ketones (Raventós et al. 2002; Ivanovs and Blumberga 2017). On the other hand, high MW (> 400 Da) or polar compounds, such as hydroxyl, carboxyl, sugars, polysaccharides, amino acids, proteins, phosphatides, glycosides, and inorganic salts, are relatively insoluble in Sc-CO_2_ (Raventós et al. 2002). Due to the low polarity nature of CO_2_, this limits its use in extracting polar bioactive compounds (Uddin et al. 2015). Polar compounds are bound very firmly to the matrix that even solvation power of pure CO_2_ may be insufficient (Anklam et al. 1998). Nevertheless, the stark difference in polarity between Sc-CO_2_ and polar compounds can be overcome by employing co-solvents. This is due to the stronger interaction between co-solvent and solute which helps increase the polarity of the solvent (Zhang et al. 2002).

Co-solvents have normally intermediate volatility between the supercritical fluid and the compound to be extracted. It facilitates the solubility of materials, such as lipid from tuna viscera (Kang et al. 2005; Ferdosh et al. 2015), phospholipids from Atlantic salmon (*Salmo salar*) (Haq and Chun 2018), carotenoids, lipids, and astaxanthin from Brazilian redspotted shrimp waste (*Farfantepenaeus paulensis*) (Sánchez-Camargo et al. 2011, 2012) in CO_2_. Furthermore, co-solvents help improve the selectivity, and solute solubility either by increasing the density of solvent or by specific chemical interaction with the solute through hydrogen bonding (Raventós et al. 2002). For example, co-solvents, such as methanol, ethanol, or water, ranging from 1 to 10% change the polarity of Sc-CO_2_ and hence increase its solvating power towards the analyte of interest, which is polar (Herrero et al. 2013). Tanaka et al. (2004) successfully recovered > 80% of the phospholipids from salmon roe when the extraction was performed by Sc-CO_2_ containing 20% ethanol as compared to only 39% without co-solvent. Zhang et al. (2002) postulated that the addition of co-solvent makes the dissolution process in Sc-CO_2_ becomes less exothermic. However, there are difficulties in separating co-solvent from the product after extraction (Raventós et al. 2002) which makes ethanol or water the most preferred co-solvent (Moyler 1993). Both co-solvents are GRAS, and environmentally benign, which can be used in the nutraceutical-related extraction process (Veggi et al. 2014). For example, ethanol is commonly used in the pharmaceutical and food industries as 50 mg of residual ethanol per day is safe for human health (Molino et al. 2018).

As shown in Table 1, it is noteworthy that lipid extraction without co-solvent was mostly identified in the production of fish oil and the recovery of enzymes and protein. However, Kang et al. (2005) reported milder application of pressure (10.3 to 13.8 Mpa) and temperature (25 to 40 °C) for a relatively short extraction time (120 min) with ethanol as a co-solvent. They recovered up to 50% of the protein content from the defatted tuna viscera at 12.4 Mpa and 35 °C for 60 min. Nevertheless, it is difficult to postulate and compare the effect of co-solvent in Sc-CO_2_ on the protein properties and enzyme activities due to lack of data.

**Table 1:** An overview of the sample preparation and Sc-CO_2_ parameters for fish oil and enzyme recovery

Sample	Particle size (µm)	Sample: CO_2_ mass ratio	Sc-CO_2_ parameter				Oil yield (%)	Protein yield/ Total protein	Enzyme activity	References
			Pressure (MPa)	Temperature (°C)	Extraction time (min)	Flow rate (g/min)				
*Fish/ FBP oil, protein properties and enzyme recovery studies*										
Tuna viscera	250–1000	-	10.3–13.8	25–40	20–120	50	58–97	38–50%	NA	Kang et al.(2005)
Squid viscera	710	1:3	25	35–45	5–40	3	28–33	NM	NM	Park et al. (2006)
Mackerel viscera	710	1: 3	25	35–45	24	3	13–16	NM	Protease, lipase, amylase: 67.3- 84.1%	(Park et al. 2008)
Squid viscera	700	1:4	15–25	35–45	150	22	22–30	NM	Protease, lipase, amylase: 0.062 -0.4 U/ml	Uddin et al. (2009)
Starfish pyloric caeca	NM	1:4	25	40	150	28	NM	Phospholipase A2: 3978 mg/ml	Phospholipase A2: 100 U/mg	Chun et al., (2010)
Mackerel viscera	NM	1:4	25	45	150	28	NM	Trypsin: 1390 mg/ml	Trypsin: 0.8 U/mg	Chun et al. (2011)
Krill	700	1:2	15–25	35–45	150	22	NM	NM	Protease, lipase, amylase: 0.05–0.45 U/mg	Ali-Nehari et al. (2012)
Mackerel muscle	NM	1: 5	15–25	45	120	27	16–20	9.28 mg/ml	Lipase, protease, Trypsin, amylase: 8- 45 U/mg	Asaduzzaman & Chun (2015)
Gilthead sea bream	NM	-	25	45	240	167	NM	73 mg/ml	Proteolytic and Trypsin activity: 0.0057 & 0.0116 U/mg	Lamas (2015)
*Fish/FBP oil studies*										
Brazilian redspotted shrimp waste (head, tail and shell)	331	1:20	30	50	20	5	2.21 (without co-solvent) 2.9 (with co-solvent)	49	NA	(Sánchez-Camargo et al. 2011)
Brazilian redspotted shrimp waste (cephalothoraxes, shells and tails)	331	1:20	30	50	100	3000	39.7- 93.8 (diff. co-solvent %)	NA	NA	(Sánchez-Camargo et al. 2012)
Catfish viscera	NM	1:1	10—40	35—80	60–240	1—3	47.5–67	NA	NA	(Sarker et al. 2012)
Carp viscera	NM	1:5	20–40	40–60	30–180	3–6	2.6–74.8	NA	NA	(Lisichkov et al. 2014)
Mackerel waste (skin (S), muscle tissue (F)), and viscera (V))	200–500	1:2	35	60	360	2	F,V,S: 20–43	NA	NA	(Hajeb et al. 2015)
Tuna waste (head (H), skin (S) and viscera (V))	500–1000	1: 2	40	65	120	3	H,S,V: T. tonggol: 13.5–35.6 E. affinis: 16.1–28.4 A. thazard: 16.8–29.5	NA	NA	(Ferdosh et al. 2015)
Atlantic Salmon by-products ((belly part, trimmed muscle, frame bone and skin)	NM	1:1	25	45	180	27	76.21–86.99	NA	NA	(Haq et al. 2017)
Carp tissues: viscera (V), fillets (F) and caviar (C)	NM	1: 5	20–40	40–60	30–180	3	C,F,V: 8–51	NA	NA	(Kuvendziev et al. 2018)

*NA* Not applicable, *NM* not mentioned; “- “: The sample: CO_2_ mass ratio could not be calculated due to insufficient information in the articles

## The effect of moisture content and particle size of fish samples on lipid extraction

**Fig. 3:**
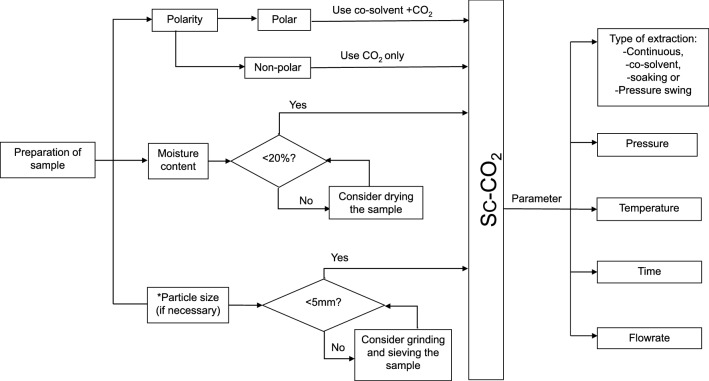
A process flow diagram for the sample preparation of FBP prior to Sc-CO_2_

The preparation of the sample is crucial prior to Sc-CO_2_ extraction. The moisture content, particle size, and the polarity of the sample of the analyte should be considered to achieve high oil extraction at minimal protein denaturation, and saving both processing time and sample usage. Figure 3 shows the overview of the moisture control, particle size, and polarity requirements for the sample preparation. The disparity in polarity between Sc-CO_2_ and food sample of the extraction process was previously addressed in supercritical carbon dioxide as a solvent for lipid extraction.

The moisture acts as a barrier against CO_2_ diffusion in the sample and the release of lipids from cells, thus, reducing the contact time between the solvent and solute (Ivanovs and Blumberga 2017). It seems that the presence of moisture in the Atlantic mackerel fillets incorporated negative interactions between oil in the supercritical phase of CO_2_ as proposed by Dunforda et al. (1998). Ivanovs and Blumberga (2017) deduced that the low oil recovery was due to high moisture content acting as a barrier to the diffusion of Sc-CO_2_ into the sample matrix and diffusion of oil out of the matrix.

Dunford et al. (1997) studied the effect of moisture content in Atlantic Mackerel (*Scomber scombrus*) on the Sc-CO_2_ extraction of oil and residual proteins. The fish samples were freeze dried to 3.8, 10.2, and 26% of moisture content from the original level of 64%. It was concluded that the oil solubility in Sc-CO_2_ decreased with increasing moisture content. Dehydration of mackerel muscle to 10.2% resulted in high oil yield with minimum changes in protein content and composition. They noticed that the amount of oil extracted was increased by 12% when moisture content decreased from 64.0 to 26.0%.

Rubio-Rodríguez et al. (2008) ascertained the negative effects of moisture on the oil extraction yield in hake (*Merluccius capensis-Merluccius paradoxus*) by-products only when the moisture is greater than 20%. The moisture content below 20% is effective to minimize the interaction between fish oil and water in the supercritical phase and hence improve the contact between fish oil and Sc-CO_2_. The sample is mostly subjected to a freeze drying process at below –40 ℃ to reduce the moisture content (Ivanovs and Blumberga 2017). Freeze drying is mostly restricted to delicate and heat-sensitive materials of high value (Azwanida 2015), and thus, appropriate for the recovery of fish protein and enzymes. Rubio-Rodríguez et al. (2008) also reported that freeze drying maintains the pore structure of the sample when compared with air drying. Air drying caused lipid pillars of the membranes to close their pores and make the membrane impermeable (Brunner 1994). Also, the addition of water-absorbing reagents, such as sodium sulphate or Hydromatrix helped control the moisture content in a sample (Anklam et al. 1998). However, the moisture content of the sample was not specifically mentioned in most studies. Sarker et al. (2012) dried the catfish viscera to achieve a moisture content of 3.95% by freeze drying the sample before Sc-CO_2_ extraction.

Reducing particle size increases the sample’s surface area, allowing Sc-CO_2_ to make better contact or penetrate to the target analyte in the solid matrix. This is due to smaller intraparticle diffusion resistance, thus, shorter diffusion path (He et al. 2003; Azwanida 2015; Khaw et al. 2017). As shown in Table 1, most of the researchers used the particle size of fish dried samples ranging from 1 to 0.2 mm for the Sc-CO_2_ extraction. Kang et al. (2005) achieved the highest extraction of lipid at 12.4 Mpa, 35 °C for 60 min from the sample with a particle size of 0.25 mm. On the contrary, Rubio-Rodríguez et al. (2008) claimed that the hake offcuts sample having particle size either more than 5 mm or less than 5 mm did not give a significant influence on the extraction yield. Although the small particle size of the sample promotes efficient extraction, there is no exact particle size requirement in fish oil extraction using Sc-CO_2_.

Nevertheless, too small particle size would hinder the fluid flow through the particles bed and decrease external mass transfer (Brunner 1994). It also causes an inconsistent flow rate of Sc-CO_2_, which consequently reduces the final efficiency of the extraction (Rubio-Rodríguez et al. 2008; Lee et al. 2020). Besides, further reduction of the particle size increased packing density in the extraction column causing the channelling effect, which influences the mass transfer rate of solvents (Said et al. 2015; Lee et al. 2020). This could be due to a strong cohesive force between particles leading to agglomeration of extraction material (Nejad-Sadeghi et al. 2015). This phenomenon is even more apparent to the pasty consistency of chopped muscle with high lipid as inferred by Dunford et al. (1997), which reduces the contact between Sc-CO_2_ and sample. Therefore, it is highly recommended that the fish muscle containing high fat content, which tends to agglomerate need to be frozen or subjected to liquid nitrogen prior to grinding into particles (Hardardottir and Kinsella 1988).

## The effects of Sc-CO_2_ on the yield of fish oil

As shown in Table 1, it was observed that the Sc-CO_2_ conditions for simultaneous recovery of fish oil, protein, and enzyme were milder than that for fish oil alone. The range of pressure and temperature is 10.3–25 Mpa and 25–45 °C, respectively, for protein and enzyme recovery study. The extraction time varies from 20 to 240 min with a flow rate between 3 and 167 g/min. Meanwhile, the temperature and pressure used for a standalone extraction of fish oil varied from 10 to 40 Mpa and 35 to 80 °C, respectively, with a much longer extraction time of 30–360 min. Furthermore, the flow rate of CO_2_ used for a standalone extraction of fish and FBP oil was higher ranging from 1 to 3000 g/min.

Kuvendziev et al. (2018) emphasized that there is a direct impact of operating pressure and temperature on the physical properties of Sc-CO_2_. This is because these two parameters have a direct impact on the vapour pressure of the solutes with the density and viscosity of the Sc-CO_2_ as mentioned by Lisichkov et al. (2014). Sc-CO_2_ pressure seems to exert more effect on the yield of fish oil as compared to temperature, extraction time, and CO_2_ flow rate. It was postulated that an increase in pressure at constant temperature increased the density of Sc-CO_2_, and hence the solvating power (Uddin et al. 2009; Asaduzzaman and Chun 2015). This is also supported by Hawthorne et al. (1992) in which the total oil yield might improve once the solubility of the oil in Sc-CO_2_ is enhanced by elevating the pressure at a given temperature. Sarker et al. (2012) reported that the pressure changes (10 to 40 Mpa) on oil yield had a more noticeable and significant effect than that of temperature (35 to 80 °C). They observed that the total oil yield extract (59.8–64.4%) increased at relatively high pressure (more than 28 Mpa) and medium to high temperature from 55 to 65 °C. However, the oil yield started to gradually decrease with pressure when the temperature was above 65 °C due to the reduction of CO_2_ density and solvation power of the Sc-CO_2_ (Sarker et al. 2012).

Moreover, Lisichkov et al. (2014) reported that the increase of pressure (30 to 35 Mpa) would increase the yield of fish oil (2.70 to 34.13%) due to the increase of the CO_2_ density at 40 °C, with a constant CO_2_ flow rate of 3 g/min. Asaduzzaman and Chun (2015) examined the effect of pressure (15–25 Mpa) at 45 °C on the oil yield from mackerel muscle and reported that the highest oil yield of 20% was obtained at 45 °C and 25 Mpa for 2 h. The effect of pressure can be attributed to the increase in solvent power and by strengthening the intermolecular physical interactions between the solvent and solute.

The effect of pressure on oil yield is due to an increase in CO_2_ density and hence, increased lipid solubility in Sc-CO_2_ (Temelli 2009). However, the effect of temperature on the yield of fish oil is not well elucidated (Sarker et al. 2012). This may be well due to more competing parameters involved. For example, an isobaric increase in temperature would decrease the CO_2_ density, while the vapour pressure of solutes increases (Temelli et al. 2005). The overall impact of these two opposing effects of temperature on solubility is dependent on the pressure, leading to the well-known crossover phenomena of solute solubility isotherms (Temelli et al. 2005; Temelli 2009). Hence, the resultant impact of temperature on solubility is dominated by whichever parameter that is greater at a given pressure. During the extraction, the mass transfer kinetics would enhance because of the diffusivity increase with temperature. At constant CO_2_ density, an increase in temperature would increase the solubility because of an exponential increase in the solute vapour pressure (Temelli et al. 2005).

Lisichkov et al. (2014) and Kuvendziev et al. (2018) investigated the influence of temperature within the range of 40 to 60 °C at a constant CO_2_ flow rate. They found out that increased temperature at lower pressure from 20 to 30 Mpa had a negative influence on the mass transfer, resulting in a decrease of the total oil yield. This is due to the reduction of CO_2_ density which predominates and hence decreased the solvating power of the Sc-CO_2_. However, further increase in temperature at elevated pressures from 35 to 40 Mpa increased the extraction yield due to increased vapour pressure of solutes, thus, increasing their solubility which overpowers the previous effect. Likewise, this justification was documented in a study conducted by Sarker et al. (2012). Uddin et al. (2009) investigated the effect of temperature (35–45 °C) and pressure (15–25 Mpa) on the oil extraction of squid viscera. It was found that the highest oil extracted (30%) was at 45 °C, 25 Mpa for a duration of 2.5 h. Park et al. (2008a) reported a low oil yield at 35 °C 25 Mpa and from mackerel viscera, but it was increased from 13 to 16% when the temperature increased up to 45 °C.

Apparently, the CO_2_ flow rate had a positive effect on the oil yield. Lisichkov et al. (2014) investigated three different CO_2_ flow rate which were 3.23, 4.56, and 5.9 g/min on the oil yield at constant pressure (35 Mpa) and temperature (40 °C). They concluded that there was a significant increase in the oil yield approximately 40 to 50% when the CO_2_ flow rate was increased from 3.23 to 4.56 g/min. However, this effect was significantly smaller when they further increased the CO_2_ flow rate from 4.56 to 5.90 g/min. There seems to be an interaction between pressure, temperature, and CO_2_ flow rate on the oil yield. At constant temperature (40 °C) and extraction time (180 min.), Lisichkov et al. (2014) reported the increase of CO_2_ flow rate from 3 to 6 g/min with increasing pressure (20 to 40 Mpa) resulted in a high yield of fish oil up to 60%. Meanwhile, at constant pressure (35 Mpa) and extraction time (180 min.), they observed there was an initial increase of yield of fish oil up to 67% when they increased CO_2_ flow rate from 3 to 5 g/min with the increasing temperature increased (from 40 to 60 °C). However, there was a 10% drop in oil yield when the CO_2_ flow rate was increased from 5 to 6 g/min. This can be concluded that increased CO_2_ flow rate had a greater impact at isobaric conditions than at isothermal conditions (Lisichkov et al. 2014).

The yield of fish oil is also affected by the extraction time. The time required for Sc-CO_2_ is dependent on the amount of fat content in each fish sample. Lisichkov et al. (2014) calculated the yield of fish oil at different extraction times (0 to 180 min) and pressure (20 to 40 Mpa) at a constant temperature of 60 °C and CO_2_ flow rate of 6 g/min. They found out that after 180 min, the extraction process entered the equilibrium state and the dynamics curve reached a plateau. The same trend of extraction time was also discovered by Kuvendziev et al. (2018) except they conducted at a constant temperature of 40 °C and CO_2_ flow rate of 3 g/min. Therefore, both studies suggested that the ideal extraction time for Sc-CO_2_ to be conducted would be between 150 to 180 min. Between those extraction time, both Lisichkov et al. (2014) and Kuvendziev et al. (2018) managed to get the highest yield of fish oil (25% and 37%, respectively) at 40 Mpa with their respective constant temperature and CO_2_ flow rate. Park et al. (2008a) claimed that it is sufficient to extract oil (16%) from mackerel viscera oil for 24 min at a flow rate of 3 g/min.

It seems that a higher yield of fish oil obtained is associated with the extraction time regardless of the solvent-to-feed ratio. As shown in Table 1, Park et al. (2008a) reported 17% less oil yield as compared to that obtained by Park et al. (2006) although both had the same solvent-to-feed mass ratio of 3:1. This may be well due to the shorter extraction time of 24 min reported in Park et al. (2008a) than that in Park et al. (2006) (40 min). Nevertheless, Ferdosh et al. (2015) obtained slightly lower oil yield (16.1%) from mackerel viscera after 120 min as compared to that reported by Hajeb et al. (2015) (25% for 360 min) albeit both having the same solvent-to-feed ratio of 2:1.

Sc-CO_2_ is also capable of reducing the contamination of toxic elements in fish oil for human consumption. Hajeb et al. (2015) proved that Sc-CO_2_ eliminated mercury, cadmium, and lead by almost 100% in fish oil from mackerel fish (*Rastrelliger brachysoma*) by-products at 35 Mpa, 60 °C for 6 has compared to Soxhlet extraction, wet reduction, and enzymatic extraction. These conventional methods showed high toxic elements in the extracted oil exceeding the accepted limits of 0.1 µg/g as outlined by The Global Organization of EPA and DHA Omega-3 s (GOED) (John et al. 2013). Hajeb et al. (2014) reported the optimum conditions of 61 Mpa and 39.8 °C for 4 h at a flow rate of 3.7 ml/min reduced the level of lead, cadmium, arsenic, and mercury by more than 95% in mackerel fish oil. Furthermore, the PUFAs content was indeed 2% higher as compared to that with Soxhlet extraction.

## The effects of Sc-CO_2_ on digestive enzyme activity

Comparison on digestive enzymes activity has been reported between defatted sample obtained using Sc-CO_2_ and the organic solvents. Acetone is a major organic solvent that is commonly used by researchers for lipid removal in the extraction of crude enzyme from FBPs (Park et al. 2002; El‐Beltagy et al. 2005; Kishimura et al. 2006; Jellouli et al. 2009; Khaled et al. 2011; Sila et al. 2012; Geethanjali and Subash 2013, 2018; Chaijaroen and Thongruang 2016; Shalaby et al. 2016; Zamani and Benjakul 2016; Poonsin et al. 2017; Murthy et al. 2018; Ideia et al. 2020). Chun et al. (2011) compared the effect of Sc-CO_2_ (25 Mpa, 45 °C, and 2.5 h) on the total activity of trypsin extracted from mackerel viscera with acetone reported by Kishimura et al. (2006). It was observed that the specific activity of the crude enzyme subjected to Sc-CO_2_ (0.8 U/mg) is almost the same as that using acetone (0.90U/mg). The difference in the total activity of trypsin was due to the variation of the specimens and the different defatting processes used, such as Sc-CO_2_ and acetone. Chun et al. (2011) also reported that the fish trypsin was recovered without significant denaturation after lipid removal at 25 Mpa and 45 °C for 2.5 h. The enzyme recovery of 50% was obtained after several purification processes with specific activity increment by 48-fold starting from the crude extract. This may be well explained by the mild Sc-CO_2_ conditions to selectively extract lipid without affecting the protein (Vaquero et al. 2006; Chun et al. 2011).

Park et al. (2008a) reported that the average enzyme activity of protease and lipase in Sc-CO_2_ treated mackerel was 30%, while α-amylase activity was 75% higher than that of chloroform. Furthermore, there was also a 2% increase in free amino acids after Sc-CO_2_ lipid extraction. Likewise, Asaduzzaman and Chun (2015) characterized water-soluble digestive enzymes, such as amylase, lipase, trypsin, and protease of mackerel (*Scomber japonicus*) muscle defatted by Sc-CO_2_ and hexane extraction. It was reported that the Sc-CO_2_ treated sample contained 3 and 4% more protein than the hexane-extracted sample and untreated sample, respectively. Apparently, the lowest protein content of the untreated sample was due to the interference of lipid in the mackerel muscle which made it less accessible to water. Generally, the enzyme exhibited higher activity for Sc-CO_2_ treated sample starting from amylase (45 U/mg), trypsin (10 U/mg), and lipase (8 U/mg) as compared to that of hexane extraction (amylase: 38 U/mg; trypsin: 7.8 U/mg; lipase: 6 U/mg), except for protease (Sc-CO_2_ treated sample: 19 U/mg; hexane treated sample: 22 U/mg). The reduction of protein concentration and loss of digestive enzymes activity of hexane extraction were due to the denatured protein as a result of long extraction time and the use of an organic solvent. This assured that the removal of lipids with conventional organic solvents causes protein denaturation (Park et al. 2008; Chun et al. 2011; Asaduzzaman and Chun 2015).

In contrast, Uddin et al. (2009) and Ali-Nehari et al. (2012) reported that the enzyme activity from Sc-CO_2_ (25 Mpa, 45 °C) treated squid viscera (protease, amylase, lipase: 0.062–0.58 U/ml) and krill (protease, amylase, lipase: 0.05–0.45 U/mg) was slightly lower than that of hexane (squid- protease, amylase, lipase: 0.07–0.7 U/ml; Krill-protease, amylase, lipase: 0.062–0.6 U/mg) and acetone (krill- protease, amylase, lipase: 0.06–0.5 U/mg) extraction. The digestive enzyme activities of squid viscera, krill, and mackerel viscera might be lost due to the formation of covalent complexes called carbamates with free amino groups on the enzyme’s surface as a result of interaction between CO_2_ with the enzyme. The carbamates result in charge removal at lysine residues and thus, could partially inhibit the enzyme activity (Kamat et al. 1992, 1995; Habulin and Knez 2001; Park et al. 2008).

## The effects of Sc-CO_2_ on pH and temperature stability of digestive enzymes

There is no doubt that Sc-CO_2_ is capable of removing lipid without protein denaturation and loss of enzyme functional properties, unlike organic solvents. Apart from that, Sc-CO_2_ also maintains the pH and temperature stability of the enzymes. The enzymes recovered from Sc-CO_2_ showed slightly higher or similar pH and temperature stability as compared to those treated with the organic solvents. For example, Ali-Nehari et al. (2012) reported that both Sc-CO_2_ treated and n-hexane treated samples showed the pH stability protease, lipase, and amylase within the range of 8–10, 7–9, and 6–8, respectively. Meanwhile, the optimum pH of these enzymes is within the range of 7–9. Moreover, the optimum temperature of protease, lipase, and amylase extracted from Sc-CO_2_ and hexane treated krill was 60 °C, 50 °C, and 37 °C, respectively. The activities of these enzymes were maintained at more than 80% when subjected to their respective optimum temperature before it started to decline sharply. These findings were similar to those reported by Uddin et al. (2009) and Asaduzzaman and Chun (2015).

Asaduzzaman and Chun (2015) also studied the characteristics of trypsin at the optimum pH and temperature of 10 and 30 °C, respectively, with pH stability ranging from 8 to 10. The activity of trypsin was retained at 80% up to 40 °C but rapidly deteriorated above 40 °C. According to Chun et al. (2011), the optimum temperature of mackerel trypsin after Sc-CO_2_ extraction was higher than that reported by Asaduzzaman and Chun (2015) which was 60 °C and 30 °C, respectively. Meanwhile, both studies discovered the optimum pH of trypsin from the defatted sample using Sc-CO_2_ extraction was between 8.0 and 8.5. Interestingly, Chun et al. (2011) revealed that trypsin obtained from the Sc-CO_2_ treated sample was notably stable in the presence of CaCl_2_. They ascertain that trypsin was stabilized in the presence of CaCl_2_ at 30 °C and pH 8 for 8 h, but it was inhibited by the presence of soybean trypsin inhibitor and N-p-tosyl-L-lysine chloromethyl ketone. This shows that the characteristic of trypsin from Sc-CO_2_ treated sample is similar to that reported by Klomklao et al. (2011) and Khandagale et al. (2015), who believed this was due to the trypsin primary calcium-binding site as it exhibited a higher affinity for calcium ion (Kossiakoff et al. 1977). The occupancy of the primary calcium-binding site stabilized trypsin towards thermal denaturation or autolysis (Kossiakoff et al. 1977).

## The effects of Sc-CO_2_ on protein denaturation of fish and FBP

Protein denaturation is commonly visualized and evaluated using sodium dodecyl sulphate polyacrylamide gel electrophoresis (SDS-PAGE). Park et al. (2008a) reported that there was an almost identical band intensity of electrophoretic pattern for both Sc-CO_2_ treated and untreated mackerel viscera sample, with a MW of 29 kDa when tested with SDS-PAGE. This is probably due to the mild conditions of Sc-CO_2_ (40 °C, 25 Mpa) during lipid extraction that the denaturation of protein was minimized. Similar findings were also reported by Uddin et al. (2009) between untreated, Sc-CO_2_ treated, and hexane treated samples of squid viscera. This scenario is interrelated with enzymes structure and its activity as described by Wimmer and Zarevúcka (2010) in which the enzymes’ three-dimensional (3D) structure can be significantly changed, resulting in denaturation and loss of activity under rigorous conditions of Sc-CO_2_. Most interestingly, the increase in depressurization cycle during Sc-CO_2_ extraction of lipid decreased the enzyme activity due to alteration of the enzyme 3D structure (Wimmer and Zarevúcka 2010). Asaduzzaman and Chun (2015) observed prominent protein bands in water-soluble extracts of Sc-CO_2_ treated sample indicating no denaturation of protein as compared to hexane treated sample. Chun et al. (2011) inferred that trypsin was found to be homogenous on SDS-PAGE and appeared as a single band with MW of 24 kDa. They stipulated that Sc-CO_2_ effectively removed lipids while causing insignificant denaturation of fish trypsin. It was also reported that the purification technique using only two steps of gel filtration is effective in getting purified trypsin as shown in their electrophoretic pattern.

## Conclusion

The strong binding of lipid to protein tissue may hinder the efficiency of the purification process of proteolytic enzymes. It is proven that Sc-CO_2_ able to remove lipid from fish and FBPs with minimal denaturation of protein for high quality fish oil and better enzyme activity due to its milder extracting conditions. Nevertheless, a clear assessment on the commercial advantage of this green technology against others should be given more attention among the industrial players. The fish sample preparation prior to fish oil extraction and optimization of Sc-CO_2_ parameters, such as pressure, temperature, time, and flow rate should be given prominence without compromising the enzyme activity. The base process scheme which only requires extraction and separation in Sc-CO_2_ technology should be feasible to be scaled up for commercialization. Sc-CO_2_ technology would be compatible or even superior to the conventional extraction method through understanding of the process fundamentals considering the phase equilibria of the mixtures involved, the development of process design, and mathematical models that are able to predict the extraction process.

## Data Availability

Not applicable. Data sharing are not applicable to this article as no datasets were generated or analysed during the current study.

## Abbreviations

et al*.*: And others
BCC: Business Communication Company
BSE: Bovine Spongiform Encephalopathy
CaCl_2_: Calcium chloride
CO_2_: Carbon dioxide
°C: Degree Celsius
Da: Dalton
DHA: Docosahexaenoic acid
t_ext_: Extraction time
EPA: Eicosapentaenoic acid
FBPs: Fish by-products
F: Flow rate
g/min: Gram/minute
GC: Gas Chromatography
GOED: Global Organization of EPA and DHA
GRAS: Generally regarded as safe
h: Hour
HCl: Hydrochloric acid
kDa: Kilo Dalton
LAHPO: Linoleic acid hydroperoxides
LC: Liquid chromatography
Mpa: Megapascal
Mg: Milligram
Mm: Millimetre
min.: Minute
MMT: Million Metric Tonnes
MW: Molecular weight
Mm: Micrometer
%: Percentage
PUFAs: Polyunsaturated fatty acids
P: Pressure
P_C_: Critical pressure
ROS: Reactive oxygen species
Sc-CO_2_: Supercritical carbon dioxide
SDS-PAGE: Sodium dodecyl sulphate polyacrylamide gel electrophoresis
SFC: Supercritical fluid chromatography
SFC-SMB: Supercritical fluid chromatography with simulated moving beds
3D: Three dimensional
T: Temperature
T_C_: Critical temperature
U/mg: Unit/ milligram
U/ml: Unit/millilitre
